# Using Citizen Science to examine health and access to health care of uninsured patients in Germany

**DOI:** 10.1093/eurpub/ckac131.104

**Published:** 2022-10-25

**Authors:** J Warth, R Köllges, L Böckenholt, M Kriegsmann-Rabe

**Affiliations:** 1 Institute of General Practice, Centre for Health, Medical Faculty of the Heinrich Heine University, Düsseldorf, Germany; 2 Anonymer Krankenschein Bonn e.V., Association for Medical Care for People without Health Insurance, Bonn, Germany

## Abstract

**Background:**

Estimates suggest that numerous people live without health insurance in Germany. Existing evidence on uninsured patients’ health and care use is scarce, specifically in Germany. The present study involves citizens’ engagement to identify community perceptions of factors associated with health and medical care and to generate community-driven policy recommendations.

**Methods:**

Representatives of civil society, affected patients and citizens (‘co-researchers') participate in a participatory health research project (MoveCitizenS) located in Bonn, Germany, using Photovoice and Community-based Mapping. The study is composed of five work packages over a 24-month period: (1) Photovoice workshops for co-researchers to produce, select and analyse photographs; (2) a series of workshops to conduct community-based mapping to identify barriers and facilitators of health care utilization; (3) workshops to co-design a cross-sectional survey of uninsured patients (n = 300); (4) project evaluation by co-researchers; (5) a dissemination strategy (e.g. advocacy event, exhibition) will be developed.

**Results:**

Procedures to facilitate the active engagement of citizens and patients are discussed. Preliminary results on community perceptions of uninsured patients’ health and medical care and factors influencing health outcomes and care utilization are presented at the conference.

**Conclusions:**

This is the first citizen science study which facilitates the understanding of barriers and enabling factors of good health and access to medical care for patients who lack health insurance coverage in Germany. This case study of a participatory project can be adapted to a range of settings to integrate local perspectives to improve population health for all.

**Key messages:**

• Numerous patients in Germany are uninsured but evidence on their health and medical care is lacking.

• Using participatory methods to address these key questions is an important approach to generate suitable policy recommendations to meet the needs of the community and improve population health.

